# Effectiveness of Pre-discharge Educational Intervention Session in the Prevention of Arm Lymphedema Among Post-mastectomy Women in a Teaching Hospital in Bangalore, India

**DOI:** 10.7759/cureus.41335

**Published:** 2023-07-03

**Authors:** Malarvizhi K Natarajan, Nalini S J, Jaya Mohanraj, Usha Vishwanath

**Affiliations:** 1 Cardiovascular Nursing, HOSMAT College of Nursing, HOSMAT Hospital Educational Institute (HHEI), Bangalore, IND; 2 Obstetrics and Gynaecology Nursing, Sri Ramachandra Institute of Higher Education and Research (SRIHER), Chennai, IND; 3 Community Health Nursing, HOSMAT College of Nursing, HOSMAT Hospital Educational Institute (HHEI), Bangalore, IND; 4 Obstetrics and Gynaecology, Sri Ramachandra Institute of Higher Education and Research (SRIHER), Chennai, IND

**Keywords:** pre-discharge educational interventions, demonstration of self/simple lymphatic drainage and exercise, post-mastectomy, breast cancer, arm lymphedema, risk reduction practices, prevention

## Abstract

Background: Lymphedema is a severe post-mastectomy complication that still causes much morbidity in breast cancer patients with axillary lymph node dissection. Therefore, after mastectomy, lymphedema prevention is crucial for long-term survival and an increase in quality of life.

Aim: The study's primary objective was to determine whether pre-discharge educational intervention sessions for post-mastectomy women effectively improve the knowledge in preventing arm lymphedema.

Methodology: A quasi-experimental research study, one-group pretest-posttest design, was conducted in a teaching hospital in Bangalore. The sample included 80 females who were diagnosed with breast cancer, had recently undergone mastectomy, and had chemo and radiation therapy plans. Questions were based on the knowledge of lymphedema preventive concepts, including the definition of the lymphatic system and lymphedema, its causes, symptoms, prevention, and management, and were included in a structured self-administered questionnaire. Convenience sampling was used among women who had undergone mastectomy for breast cancer. The pre-discharge educational interventions session included instructions on a self or simple lymphatic drainage technique demonstration, arm exercises, and an e-brochure on risk reduction strategies and arm lymphedema prevention. The knowledge of pre-discharge educational intervention sessions highlighting risk reduction/prevention strategies among post-mastectomy women was evaluated using the self-structured knowledge questionnaire pretest and posttest data.

Results: The entire study population comprised women who had undergone mastectomy. Almost half of the subjects were older than 55 years. Prior to the intervention, the majority of patients (58) had poor knowledge (72%) about preventing lymphedema, whereas nearly all patients (80) had good knowledge (100%) after the intervention sessions. All participants felt comfortable using the treatment plan to avoid arm lymphedema. The knowledge gain was statistically significant at the 0.05 level.

Conclusions: It was determined that pre-discharge educational intervention sessions enhanced post-mastectomy women's awareness and risk reduction behaviors toward preventing arm lymphedema and reducing arm morbidity. Therefore, it is suggested that women who have had breast cancer surgery participate in a pre-discharge educational intervention program. This will guarantee that all mastectomy patients have access to educational information/materials and that risk reduction strategies are followed to prevent lymphedema.

## Introduction

The leading cause of cancer among women in India is breast cancer, followed by cervical cancer. About 6,85,000 people died in 2020 due to breast cancer worldwide, which affected 2.3 million women [1]. The National Center for Disease Informatics and Research's report on the National Cancer Registry Program predicted that more than two lakh women in India would have been diagnosed with breast cancer by 2020, with a mortality rate of 37.2% and more than 76,000 reported fatalities. In 2025, more than 2.3 lakh cases will likely be in India. According to the Indian Council of Medical Research (ICMR) figures, the percentage of breast cancer diagnoses in Karnataka increased from 16% per 100,000 in 1998 to 34% per 100,000 in 2008. According to the population-based cancer registry (PBCR) report 2013, Bangalore city tops the list with 36.6 new instances for every one lakh people with the condition [2].

When breast cancer is detected early, treatment for the disease can be very successful, with a survival probability of 90% or higher. However, modified radical mastectomy (MRM) is still a typical surgical surgery for breast cancer, particularly in underdeveloped nations. The entire breast, including the nipple, areola, skin, subcutaneous fatty tissue, and axillary lymph nodes, must be removed [3]. Therefore, lymphatic system disorders are brought on by conventional therapies like radiotherapy and surgery (axillary node dissection) [4].

One of the most common and dreaded side effects of breast cancer treatments is lymphedema, which has long-term physical and psychological effects on patients. It is characterized by an aberrant and localized protein-rich fluid buildup in interstitial space that can result in edema and long-term inflammation [5]. For breast cancer patients who underwent axillary lymph node dissection, lymphedema remains a primary cause of morbidity. Health professionals face difficulties due to the progressive nature and lack of efficient treatments [3]. The other cause for lymphoedema is antineoplastic therapy, which not only results in long-term physical therapy, psychological harm, and a decline in quality of life but also raises the danger of concomitant illnesses (such as infections, inflammations, or erysipelas) [6]. Chronic swelling, localized pain, atrophic skin changes, and secondary infections are some of its clinical characteristics [7]. Once it manifests, lymphedema linked to breast cancer is a lifelong problem. Since there is no cure for lymphedema, treatment objectives are to reduce uncomfortable symptoms and minimize inflammation.

Complete decongestant therapy (CDT), arm exercises, manual lymphatic drainage (MLD), skin and nail care, self-massage, compression bandages, and/or hand sleeves are some of the techniques used to treat post-mastectomy lymphedema [8]. Through patient education about signs and symptoms and early identification, nurses play a crucial part in preventing lymphedema following mastectomy. All breast cancer patients should undergo routine evaluations and be asked about any swelling, reduced range of motion in the extremities, stiffness in the shoulder joint, and other lymphedema symptoms [6]. Arm exercises are essential to rehabilitating mastectomy patients because they increase muscle strength and upper limb function, lessen pain and suffering, and enhance confidence [3]. In addition, the National Lymphedema Network (NLN) recommends several precautionary measures, including avoiding aggressive skin care to prevent trauma or injury (avoid needle sticks, blood withdrawals from the affected arm, or intravenous sticks), avoiding constriction of the limbs (blood pressure cuff inflation and tight clothing), avoiding extreme temperatures, and frequently wearing compression garments, especially when flying [9]. Compression bandaging, aerobic or resistance exercise, self-applied MLD, intermittent pneumatic compression therapy (IPCT), the elevation of the affected extremity, and weight management are some self-care techniques for breast cancer-related lymphedema (BCRL) patients [10].

Need for the study

Around 30% of women with breast cancer (BC) undergoing surgical treatment in developing countries develop lymphedema. Before being discharged, patients must receive health education regarding understanding and self-care techniques linked to lymphedema prevention since BCRL is a lifelong problem and has no known cure. After lymphedema develops, treatment is substantially less effective than prevention [8].

BCRL risk can be reduced by patient counseling within the first week following surgery, followed by physiotherapy [11]. In addition, the early patient education program can reduce the likelihood of developing lymphedema following breast cancer surgery [12]. On the other hand, breast cancer patients felt they did not receive enough information on secondary lymphedema, how to recognize signs and symptoms, where to look for therapy and find doctors specializing in lymphedema, or how to perform some preventive behaviors [13].

The patient should be given this information timely and reminded regularly. According to one study, the optimal moment for receiving information from the patient's perspective was at the time of breast cancer diagnosis. It is a contentious topic since some medical professionals consider that informing patients about lymphedema before treatment can make them more stressed and anxious [13]. According to some studies, the timing of getting information should be left up to the patient's discretion [14]. Evidence indicated that breast cancer patients may not always have access to timely lymphedema prevention and management information, nor is it viewed as an urgent requirement [15]. It can be the result of clinicians underestimating this illness [16]. Literature has demonstrated that medical practitioners lack knowledge about lymphedema [17].

Nurses play a crucial part in preventing post-mastectomy lymphedema through patient education before symptoms and early detection. Arm training is crucial for rehabilitating post-mastectomy patients because it increases the strength and function of the upper limb muscles, lessens pain and discomfort, and enhances the quality of life and confidence [18].

The study was conducted in the present setting as the investigator identified a need to provide information on preventing BC-related lymphedema among post-mastectomy patients before they get discharged from the hospital to create an awareness of risk reduction and prevention aspects of BCRL. The educational materials provided to the patients before discharge will enable them to have a ready reckoner for information references.

## Materials and methods

Study design and setting

A quasi-experimental, one-group pretest-posttest approach was used in the research. The study was conducted in the oncology division of a teaching hospital in Bengaluru, Karnataka, India.

Study participants and sampling

The samples consisted of women above 35 years who were diagnosed with breast cancer, underwent a unilateral mastectomy with axillary lymph node dissection, and were expected to get radiation and chemotherapy. Eighty women were selected for the study using the convenience sampling method. The following factors were taken into consideration when selecting the patients for this study: (1) patients with unilateral breast cancer, (2) patients with breast cancer who had undergone mastectomy with axillary lymph node dissection, (3) female patients who are aged 35 years and above, (4) patients with no previous mastectomy, and (5) patients who agreed to participate in the study.

Data collection tools and techniques

A structured self-administered questionnaire created based on pertinent literature served as the study method. The tool consists of sections A and B.

Section A

Age, education, marital status, occupation, and clinical details such as the type of surgery, the side of the surgery, the dominant arm, the type of treatment, comorbid conditions, and body mass index (BMI) are included in this section.

Section B

The questionnaire on BCRL and risk reduction practices for patient knowledge is self-administered based on the pre-discharge intervention sessions covering the concepts of preventing lymphedema, including the definition of the lymphatic system and lymphedema, its causes, symptoms, and prevention and risk reduction methods.

This tool had 15 questions: BCRL information (eight questions) covering definition, causes, early signs and symptoms, stages, complications, and treatment as well as risk reduction practices to reduce BCRL (seven items). Each question had two options: one point for the correct answer and zero for the wrong answer.

Validity and reliability of tools

A panel of oncology specialists, including breast surgeons, medical oncologists, and radiation oncologists, evaluated the instrument's content validity to ensure its applicability and thoroughness. The necessary changes were made as a result. Reliability was calculated using the test-retest approach and was found to be 0.82. The tool was practical and trustworthy for carrying out research investigations.

Scoring and interpretation

Correct and wrong responses were used to determine the score. Every correct answer received a "one" score, while every wrong answer received a "zero.”

The level of BCRL knowledge was graded as follows: less than 50 - inadequate; 51-75 - moderately adequate; above 75 - adequate, and the total score was 0-100. A higher mean score reflected a higher level of knowledge.

Administration

The post-mastectomy women used the questionnaire tool twice: once before the pre-discharge intervention session (first or second postoperative period - one day prior to discharge from the hospital) and once after a pre-discharge educational intervention session (two weeks post-discharge during the follow-up visit to the oncology outpatient department).

Pre-discharge educational intervention sessions

The oncology department of a teaching hospital in Bangalore allowed the researcher, a nurse, and a certified lymphedema specialist to conduct the study after consulting with the appropriate authorities. First, a convenience sampling technique was used to choose the eligible samples. Then, patients who agreed to participate in the study signed a consent form. The research was carried out between December 2021 and November 2022.

Data Collection Method

**Figure 1:**
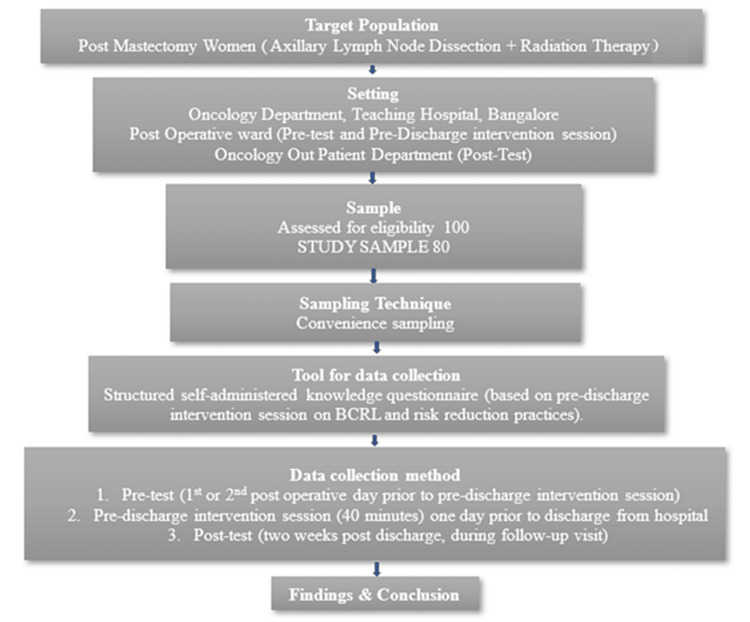
Data collection procedure

Figure 1 shows the schematic representation of the data collection procedure. On the first or second postoperative day, one day before hospital discharge, the pretest was administered utilizing a structured knowledge questionnaire. An educational intervention session on BCRL and risk reduction practices followed the pretest. This session covered information on the lymphatic system, disease conditions, aerobic exercises for the arm, self-lymphatic drainage techniques, compression garments, and risk reduction techniques based on NLN. Each patient had an individual 40-minute session with a certified lymphedema therapist nurse using live demonstration and video recording of the self-lymphatic drainage technique. An e-brochure on prevention and risk reduction aspects was given to the patients for future reference.

Posttests were conducted using the same tool two weeks after the educational session during their follow-up visit to the oncology outpatient department to gauge the understanding of lymphedema and risk reduction techniques for preventing arm lymphedema.

Ethical consideration

The Institutional Ethical Committee of HOSMAT Hospital Educational Institute (HHEI) accepted the present study, and official approval was taken (HHEI/HCON/S-1/02/2020).

Informed and written consent was gained from participants who fulfilled the inclusion criteria, and permission was secured from the administrators of the hospital's oncology department in Bangalore. The participants were completely anonymous, and confidentiality was maintained.

Analysis

The analytical method of data calculation was carried out using SPSS version 20.0 (IBM Corp., Armonk, NY). With the aid of descriptive statistics, the data was examined. First, demographic characteristics were determined using frequency and percentage. Next, the pre-and posttest knowledge score was computed using frequency, percentage, mean, and standard deviation. Finally, the pre- and posttest knowledge scores were compared using a paired t-test, which was significant at a p-value of 0.000 (p-value < 0.001).

## Results

The data were analyzed using descriptive statistics. Table 1 shows the demographic variables and clinical data of the subjects.

**Table 1:** Distribution of demographic variables and clinical data among post-mastectomy women (n = 80)

S. No.	Demographic variables	Categories	No.	%
1.	Age in years	35-44	17	21
		45-54	22	28
		55 & above	41	51
2.	Marital status	Single	1	1
		Married	68	85
		Divorced/Separated	1	1
		Widow/Widower	10	13
3.	Locality	Urban	68	85
		Semi-urban	12	15
4.	Educational status	Primary education	9	11
		Secondary education	32	40
		Graduate	22	28
		Postgraduate	17	21
5.	Occupational status	Housewife/Homemaker	56	70
		Skilled worker	6	8
		Professionals	15	21
		Retired	1	1
6.	Family income per month	Rs.10, 001–15,000	6	8
		Rs. >15,000	74	92
7.	Type of surgery	Modified radical mastectomy	80	100
8.	Lymph node dissection	Axillary lymph node dissection	80	100
9.	Side of surgery done	Right breast	42	53
		Left breast	38	47
10.	Arm at risk	Left arm	38	47
		Right arm	42	53
11.	Dominant arm	Left arm	1	1
		Right arm	79	99
12.	Comorbidity	Diabetes mellitus	7	9
		Hypertension	6	8
		Obesity	29	36
		None	38	47
13.	Body mass index (BMI)	Underweight	0	0
		Normal	39	49
		Overweight	12	15
		Obese	29	36

Section A: Sociodemographic profile of post-mastectomy women

The age group with the highest score, 55 years and older, comprised 51% of the 80 breast cancer samples evaluated. Eighty-five percent of the individuals were married, while 13% were widowed. Most of the samples (85%) resided in urban/metropolitan areas, while the remaining respondents (15%) lived in semi-urban areas. Furthermore, 11% of the subjects had a primary education, 40% had a secondary education, and 28% and 21% held graduate and postgraduate degrees, respectively. In addition, 92% of the subjects made more than Rs. 15,000 monthly. Seventy percent of the individuals were homemakers (housewives), and 21% were professionals.

Regarding clinical characteristics, almost 99% of the samples were right-handed, and about 53% had right-side mastectomy surgery. Obesity was a comorbid condition in 36% of the samples, and radiation therapy and chemotherapy were scheduled for 94%. According to BMI values, almost 51% of the participants fell into the overweight (15%) and obese (36%) categories, while 49% were normal.

Section B: Pretest and posttest knowledge scores of post-mastectomy women

**Figure 2:**
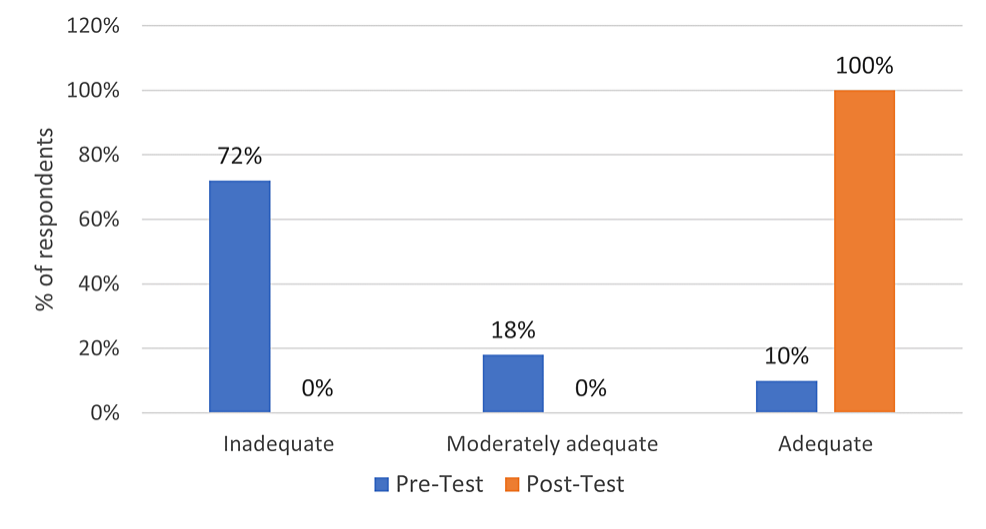
Level of knowledge

Table 2 demonstrates that in the pretest, 72% of respondents had inadequate knowledge of BCRL, while 18% had only moderately adequate knowledge. However, in the posttest, it was found that 100% of the samples had adequate knowledge of BCRL and risk reduction techniques (Figure 2).

**Table 2:** Knowledge scores among post-mastectomy women (n = 80)

S. No	Score	Level of knowledge	Pretest		Posttest	
			Frequency	%	Frequency	%
1.	Less than 50	Inadequate	58	72	0	0
2.	51–75	Moderately adequate	14	18	0	0
3.	Above 75	Adequate	08	10	80	100
Total score			0-100		0-100	

**Figure 3:**
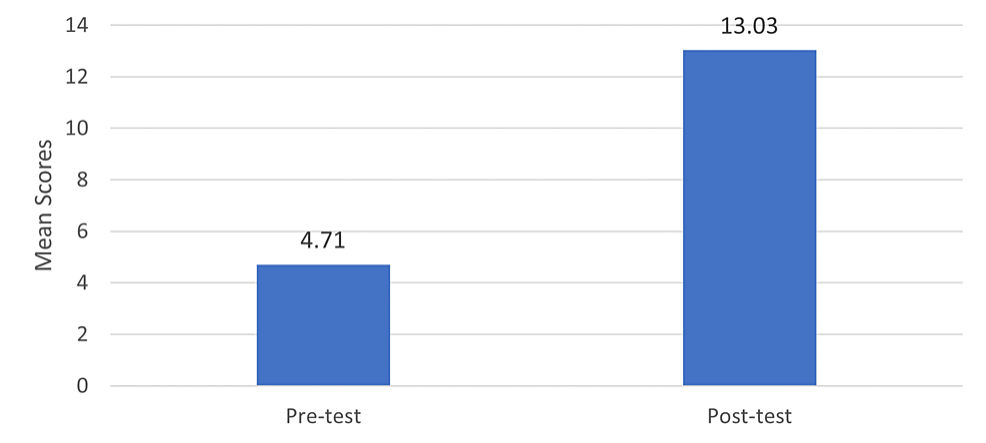
Comparison of pretest and posttest among post-mastectomy women

The mean SD of the pretest score is 4.71 ± 3.98, and the mean SD of the posttest score is 13.03 ± 0.42 as shown in Table 3. The difference between the pre- and posttest showed a significant improvement in the posttest score compared to the pretest score, which is statistically significant (t = 18.68, p = 0.000), evidenced by p-value < 0.001 (Figure 3).

**Table 3:** Comparison of pretest and posttest knowledge scores (n = 80) * denotes significance (p < 0.001).

Test	Mean	SD	t-value	p-value
Pretest	4.71	3.98	18.68*	0.000*
Posttest	13.03	0.42		

**Figure 4:**
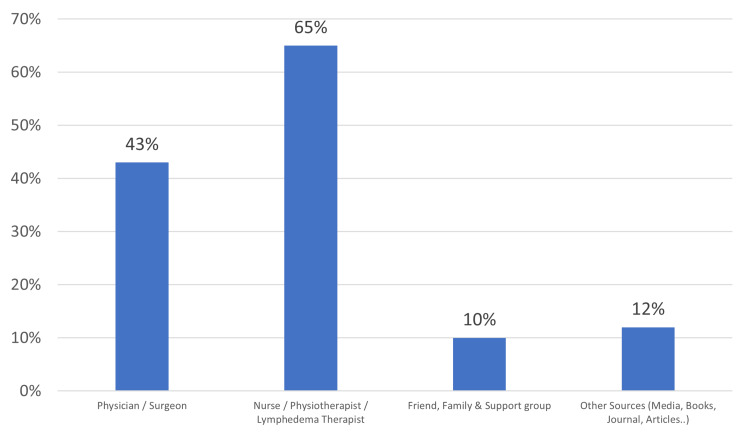
Post-mastectomy women’s sources of information on BCRL BCRL: Breast cancer-related lymphedema.

Figure 4 shows that 65% of the studied women acquired information about BCRL from healthcare professionals, specifically from nurses, physiotherapists, and lymphedema therapists.

## Discussion

BCRL is a frequent yet underreported side effect of treating breast cancer. Lymphedema (LE) impairs the general quality of life and costs patients, their caregivers, and society [19].

According to Cancer Council, 2017, lifelong self-care and management are necessary for lymphedema. The nurse can thus support the patient in achieving self-care objectives and managing the challenges of the patient's lifetime risk and particular BCRL trajectory [20]. The study samples' demographic data analysis provided insight into the general traits of post-mastectomy women.

Evaluation of post-mastectomy women's knowledge of lymphedema and its risk reduction techniques

The study was done among post-mastectomy women to improve their knowledge and risk reduction measures for preventing BCRL using a pre-discharge educational intervention session. The study showed that the mean pretest score (4.71) was less than the mean posttest score (13.03). The difference between the pre- and posttest results demonstrated a statistically significant improvement in the posttest score compared to the pretest score (t = 18.68, p = 0.000). The study reported by Borman et al. found that breast cancer survivors, particularly in developing nations, are unaware of the BCRL or the dangers of BCRL, which supports this finding that breast cancer patients should be taught the fundamentals of preventive and prophylactic measures after mastectomy by an oncologist and nurses to avoid complications. Additionally, the study emphasized that early postoperative use of lymphedema preventive measures may lessen or delay the development of it [21]. Similarly, Zuther stressed the benefits of postoperative exercise and lymphedema prevention techniques for preventing lymphedema after mastectomy [22].

Individuals' sociodemographic details and health information

The current investigation results showed that 51% of the subjects were 55 years of age or older. Most of the subjects in the current study were married and homemakers, which may indicate the burden that married women bear in caring for their family, which causes their arms to be under more stress, increasing the risk of BCRL. According to the current study's findings regarding education level, 40% had only secondary education, and 70% were homemakers. This result aligns with that of So et al. as well as El Sayed and Badr, who discovered that almost half of breast cancer patients in their study were married, held a secondary degree, and were unemployed [23,24].

Taking into account the participant's clinical characteristics

According to the present study, most subjects were right dominant hands. This may indicate that most of the women in the study were married homemakers, which may have exposed them to household pesticides and detergents that increase the risk of LE. Research by Safwat et al. at Cairo University found that a statistically significant correlation between the development of upper arm LE following breast cancer treatment and tumor in the dominant arm supports this conclusion [25].

The current study's findings regarding the BMI of the investigated women showed that 15% were overweight and 36% were obese. The study by Dean et al., which found that a high BMI is a risk factor for the start of upper body BCRL, supports this finding [26].

Overall patient knowledge and sources of information about lymphedema and its risk factors

The current study indicated that the majority of women who participated were largely unaware of breast cancer, BCRL, and LE risk reduction techniques. This finding is corroborated by a study [27], which found that the pre-program general knowledge was lacking among post-mastectomy women.

The current study indicated that the primary sources of information for our subjects about BCRL and LE risk reduction techniques were healthcare professionals, namely, doctors, nurses, physical therapists, and others. A smaller number of our subjects also obtained information online, followed by other sources like family, friends, support groups, books, and journal articles.

However, in one study, it was observed that most doctors and nurses indicated they did not frequently advise women or provide written information on BCRL prevention to their patients and the extent to which women's everyday activities were affected by BCRL following diagnosis [27]. Hence, current practices followed by healthcare professionals are leading to inadequate knowledge about BCRL and LE risk reduction techniques among post-mastectomy women.

The current study's findings highlight that pre-discharge educational interventions session significantly improved knowledge levels and confidence among women who had undergone a mastectomy in applying the concepts of lymphedema prevention.

This result aligns with the study presented by Hawash, who reported that after implementing the nursing rehabilitation program, the comprehensive knowledge improved among the studied women [28].

Limitations and recommendations

The study has limitations in some areas. The study was small and had one participant group, no control group, and no randomization, so it cannot be generalized. The study used multiple posttests to determine the long-term effects of the pre-discharge educational intervention that helps delay LE onset. Breast cancer survivors can maintain the post-mastectomy quality of life by holding frequent reinforcement sessions during follow-up.

The following recommendations for future research and practice are made in light of the findings of the current study:

In Services

All healthcare professionals working in breast cancer units, including nurses caring for women with breast cancer-related LE, should participate in ongoing in-service education programs to update, acquire, and develop the knowledge, performance, and attitude necessary to manage this population of patients.

After breast surgery, there is a clear need for educational programs on LE and risk reduction techniques that provide the most current knowledge on breast cancer-related LE and self-care strategies to assist breast cancer survivors in effectively managing their disease.

In Research

Periodically, it is advised that additional research be conducted to assess the outcomes of new management strategies for patients with breast cancer-related LE. In addition, extended research studies are required to assess the effect of implementing the developed self-care instructions for post-mastectomy women at risk or with BCRL.

Based on this study's results, it is advised that a similar study be replicated with a bigger sample size and diverse teaching strategies, together with frequent reinforcement, which is crucial in enhancing breast cancer patients' understanding.

## Conclusions

Most study participants who had undergone a mastectomy for breast cancer either did not know BCRL or had misconceptions about it, indicating a lack of proper education regarding lymphedema. The study's findings showed that the posttest knowledge score was higher than the pretest knowledge score. The framework for delivering education, which should include teaching protocols related to disease duration, risk reduction, and lymphedema therapy, is required. An individualized BCRL educational session with live demonstration and provision of a video recording and e-brochure is effective in acquiring knowledge on the prevention of BCRL development. Early and continual education for future management is crucial among the survivors at high risk for developing BCRL. Application of lymphedema prevention concepts is a key concern to improve post-mastectomy women's functional status, shoulder, and arm functions, ultimately improving their quality of life throughout their survivorship.
